# Super-Enhancer–Associated nine-gene prognostic score model for prediction of survival in chronic lymphocytic leukemia patients

**DOI:** 10.3389/fgene.2022.1001364

**Published:** 2022-09-15

**Authors:** Xue Liang, Ye Meng, Cong Li, Linlin Liu, Yangyang Wang, Lianfang Pu, Linhui Hu, Qian Li, Zhimin Zhai

**Affiliations:** Department of Hematology/Hematological Lab, The Second Hospital of Anhui Medical University, Hefei, Anhui, China

**Keywords:** chronic lymphocytic leukemia, super-enhancer, prognostic model, overall survival, CLL

## Abstract

Chronic lymphocytic leukemia (CLL) is a type of highly heterogeneous mature B-cell malignancy with various disease courses. Although a multitude of prognostic markers in CLL have been reported, insights into the role of super-enhancer (SE)–related risk indicators in the occurrence and development of CLL are still lacking. A super-enhancer (SE) is a cluster of enhancers involved in cell differentiation and tumorigenesis, and is one of the promising therapeutic targets for cancer therapy in recent years. In our study, the CLL-related super-enhancers in the training database were processed by LASSO-penalized Cox regression analysis to screen a nine-gene prognostic model including TCF7, VEGFA, MNT, GMIP, SLAMF1, TNFRSF25, GRWD1, SLC6AC, and LAG3. The SE-related risk score was further constructed and it was found that the predictive performance with overall survival and time-to-treatment (TTT) was satisfactory. Moreover, a high correlation was found between the risk score and already known prognostic markers of CLL. In the meantime, we noticed that the expressions of TCF7, GMIP, SLAMF1, TNFRSF25, and LAG3 in CLL were different from those of healthy donors (*p* < 0.01). Moreover, the risk score and LAG3 level of matched pairs before and after treatment samples varied significantly. Finally, an interactive nomogram consisting of the nine-gene risk group and four clinical traits was established. The inhibitors of mTOR and cyclin-dependent kinases (CDKs) were considered effective in patients in the high-risk group according to the pRRophetic algorithm. Collectively, the SE-associated nine-gene prognostic model developed here may be used to predict the prognosis and assist in the risk stratification and treatment of CLL patients in the future.

## Introduction

Chronic lymphocytic leukemia (CLL), a mature and monoclonal CD5+ CD23+ B cell malignancy, proliferates and accumulates in the bone marrow, blood, and lymphoid nodes (Hallek et al., 2018). It is often asymptomatic in the early stage. It is often found that painless lymphadenopathy or the absolute value of lymphocytes is increased for unknown reasons. Patients have mild fatigue, fatigue, and other non-specific manifestations. Once they enter the advanced stage, they can present with weight loss, repeated infection, bleeding, and anemia in addition to systemic lymph nodes and splenomegaly. CLL cases are fewer in Asia than those in the Western world, and it is reasonable to assume that genetic and environmental factors play roles in pathogenesis (Burger, 2020). During 2014–2018, the rate of new cases of CLL was 4.9 per 100,000 per year and the median age at diagnosis is 72 years, the death rate was 1.1 according to the aforementioned survey [The Surveillance Epidemiology and End Results (SEER) Program of the National Cancer Institute. Cancer fact sheets: chronic lymphocytic leukemia (CLL). https://seer.cancer.gov/statfacts/html/clyl.html (accessed 22 September 2021)].

CLL is widely known as a heterogeneous disease that exhibits variable clinical symptoms, time-to-treatment (TTT), easily progression and difficult prognosis. CLL patients are often diagnosed with incidental findings, and the clinical course ranges from an asymptomatic, indolent disease that requires no treatment to a rapidly progressive and chemotherapy-resistant disease until death within a short period (Burger, 2020). The indications for treatment mainly include the clinical stage and symptoms of patients, and the standard therapy is chemoimmunotherapy. Unfortunately, the majority of CLL patients are too old to tolerate intensive standard chemotherapy; therefore, an effective prognostic model is needed to predict the individual clinical courses and to improve the outcome. Over the past few decades, great advances have been made in figuring out the molecular and genetic biology of CLL to identify the indicators of progression and survival. These indicators include cytogenetics, age, IGHV gene mutation status, β2-microglobulin (β2-MG), clinical-stage (RAI/BINET stage), and so forth (Bosch and Dalla-Favera, 2019). In CLL, 13q14, 11q22-23, trisomy of 12q, and 17p deletions are found in 80% of the cases. 11q22-23 and 17p deletions are associated with poor survival, whereas 13q14 deletions and trisomy of 12q have a longer TTT and survival time (Dohner et al., 2000). TP53 aberrations (Zenz et al., 2010) indicate a more aggressive disease progression and extensive drug-resistant and worse outcome, and the same role applies to IGHV genes (Damle et al., 1999) and ZAP-70 (Crespo et al., 2003). Unmutated IGHV and high-expression of ZAP-70 have a comparatively aggressive disease course too, and other relevant risk markers include expression of CD38 (Rassenti et al., 2008), CD49d (Bulian et al., 2014), lipoprotein lipase (LPL) (Prieto and Oppezzo, 2017), serum concentrations of thymidine kinase (Hallek et al., 1999), and β2-microglobulin (Hallek et al., 1996).

In this article, a SE-associated gene list was used to carry out LASSO-penalized Cox regression analysis, and construct a nine SE-associated gene prognostic model, namely, TCF7, VEGFA, MNT, GMIP, SLAMF1, TNFRSF25, GRWD1, SLC6AC, and LAG3. Meanwhile, this model was verified by testing GEO datasets and the ICGC-CLL dataset, respectively. Univariate and multivariate Cox regression analyses, and the ROC curve were analyzed to evaluate the prognostic accuracy of this nine-gene model. Moreover, the aforementioned validated steps, the role of the nine-gene prognostic model, and the nine hub genes were further explored in CLL genesis and the relationship between this prognostic model and other known risk markers, such as IGHV status, FISH abnormality, and ZAP70 expression level. It was indicated that the model demonstrated predictive power and had an expected relationship with known risk markers. In addition, an interactive nomogram based on the nine-gene risk score and clinical traits was constructed. Finally, paired pre- and post-treatment datasets were used to examine the effects of treatments on the risk score or each of the nine hub genes’ expression, and we predicted 25 clinical drugs that may be more sensitive to high-risk patients. The improved nine-gene prognostic model of this work provided a bright future for the diagnosis, disease stratification, and therapy of patients with CLL.

## Results

### Construction of a nine-gene LASSO-penalized cox regression model and validation of independent prognostic factors

**FIGURE 1:**
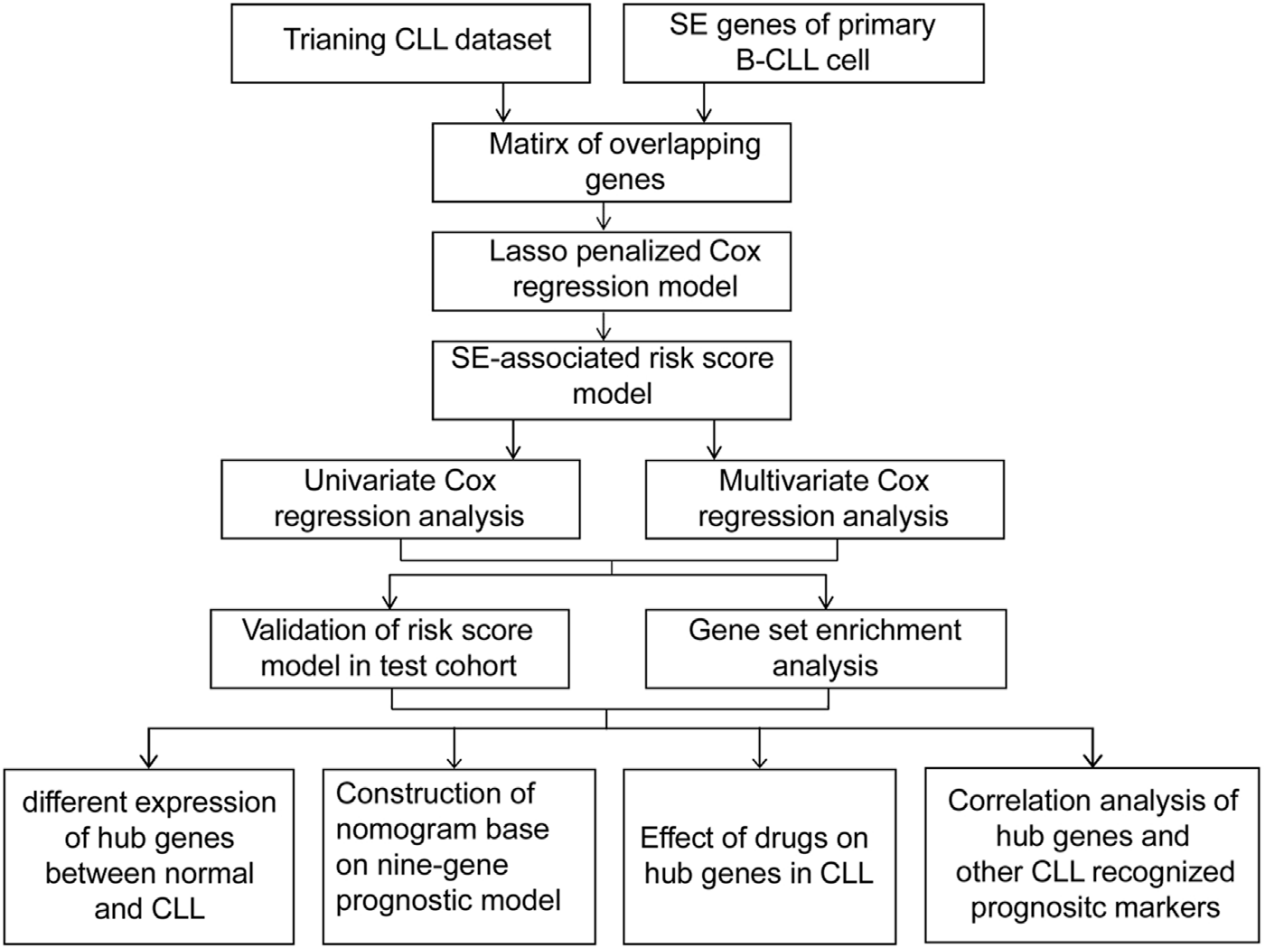
A flowchart of the overall procedure used to establish and verify the SE-associated gene-based prognostic model in CLL patients.

**FIGURE 2:**
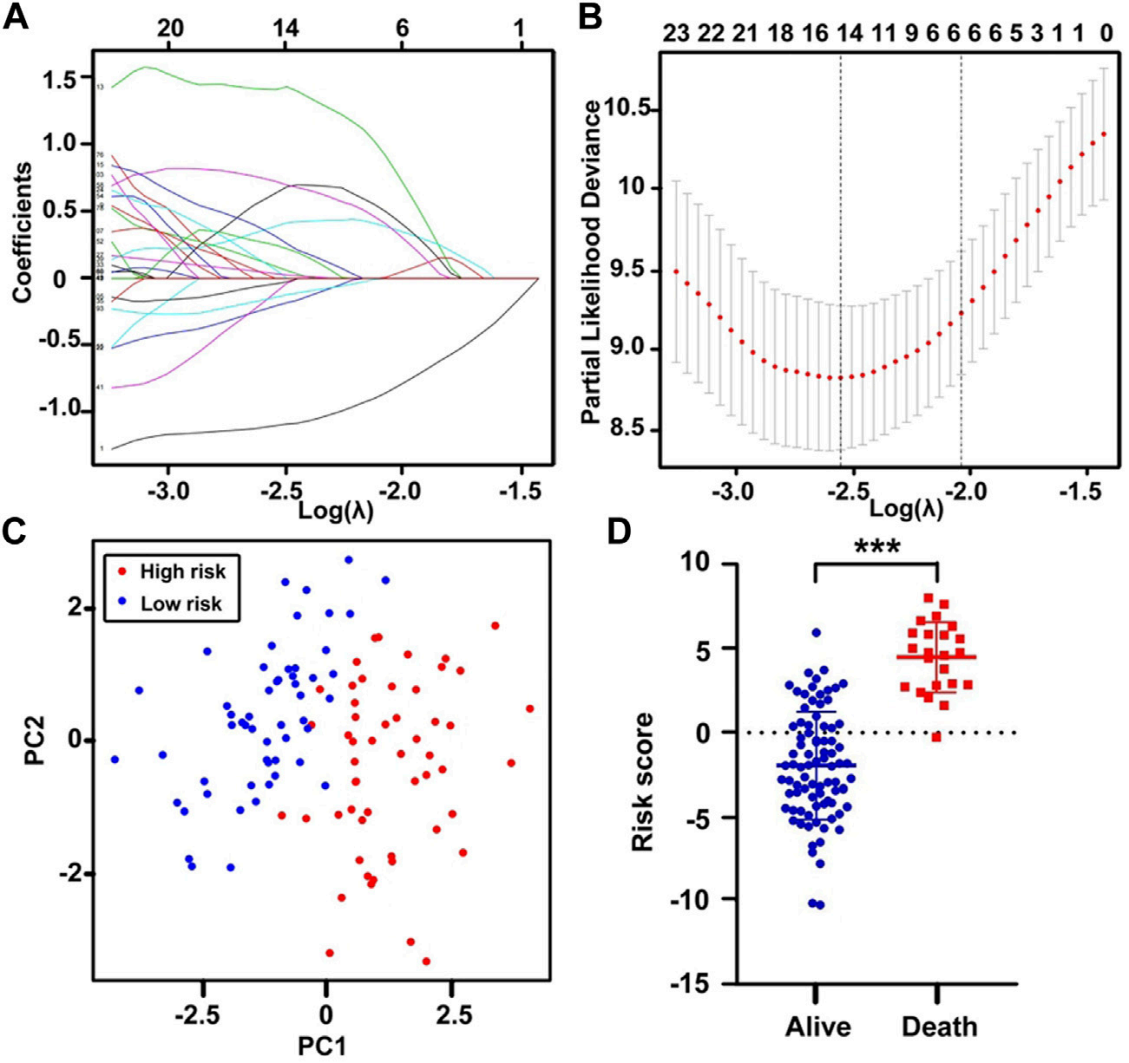
Construction of the SE-related prognostic model. **(A)** The LASSO coefficient values at various levels of penalty; each curve represents an SE-gene. **(B)** The confirmation of the best lambda value by LASSO Cox regression analysis. **(C)** Principal component analysis (PCA), red dots correspond to high-risk patients, and blue dots correspond to low-risk patients. **(D)** The scatter plot of the survival status of CLL patients based on the 9-gene model using the *t*-test. ***p < 0.001.

The flowchart featured the construction and validation of the SE-associated gene-based prognostic model of CLL and the correlation with other known risk markers (Figure 1). An 831 primary B-CLL cell-related SE list was downloaded from the website and the 18,887 gene matrix in CLL patients was provided in the GSE22762 column, and a 587 SE-associated gene matrix for CLL was gained via overlapping the aforementioned two gene sets. Immediately after, the gene matrix was done by LASSO-penalized Cox regression to screen the prognosis-related genes with potential. Figure 2A shows the coefficient values for each at various penalty levels as long as genes with non-zero coefficients had prognostic value in the LASSO-penalized regression model. Ten-fold cross-validation obtained the maximum lambda value, and we selected one model which produced a group of nine genes (Figure 2B). Principal component analysis (PCA) showed high-risk patients separate from low-risk ones evidently (Figure 2C). Also, the obvious distinction between survival and death was calculated by using the nine gene–based prognostic model, implying that the prognostic model functioned smoothly in the prediction on the OS of patients with CLL (Figure 2D).

To validate the LASSO-penalized Cox regression model, univariate Cox proportional hazard regression analysis determined that these genes affected the OS of patients with CLL independently, and all log-rank *p*-values of the nine genes were < 0.01 (Supplementary Figure S1A). Multivariate Cox proportional hazard regression analysis was also performed, and the global *p*-value of our model was only 2.64e-16 (Supplementary Figure S1B), with an AIC of 124.96 and a C-index of 0.95. These indexes suggest that the nine genes are possibly prognostic markers for OS in CLL patients. Meanwhile, the results of K–M survival analysis showed that GRWD1, SLC6A3, and MNT had no significant association with survival (Supplementary Figure S2). Furthermore, we concluded that SLAMF1, TCF7, TNFRSF25, MNT, and VEGFA were protective factors, whereas GRWD1, SLC6A3, GMIP, and LAG3 appeared to be harmful factors in CLL, based on the aforementioned hazard ratios of univariate and multivariate regression analyses. Thus, the nine-gene SE-associated model by LASSO-penalized Cox regression possibly predicted the OS of CLL patients.

### Establishment and validation of the nine gene-based risk score model

**FIGURE 3:**
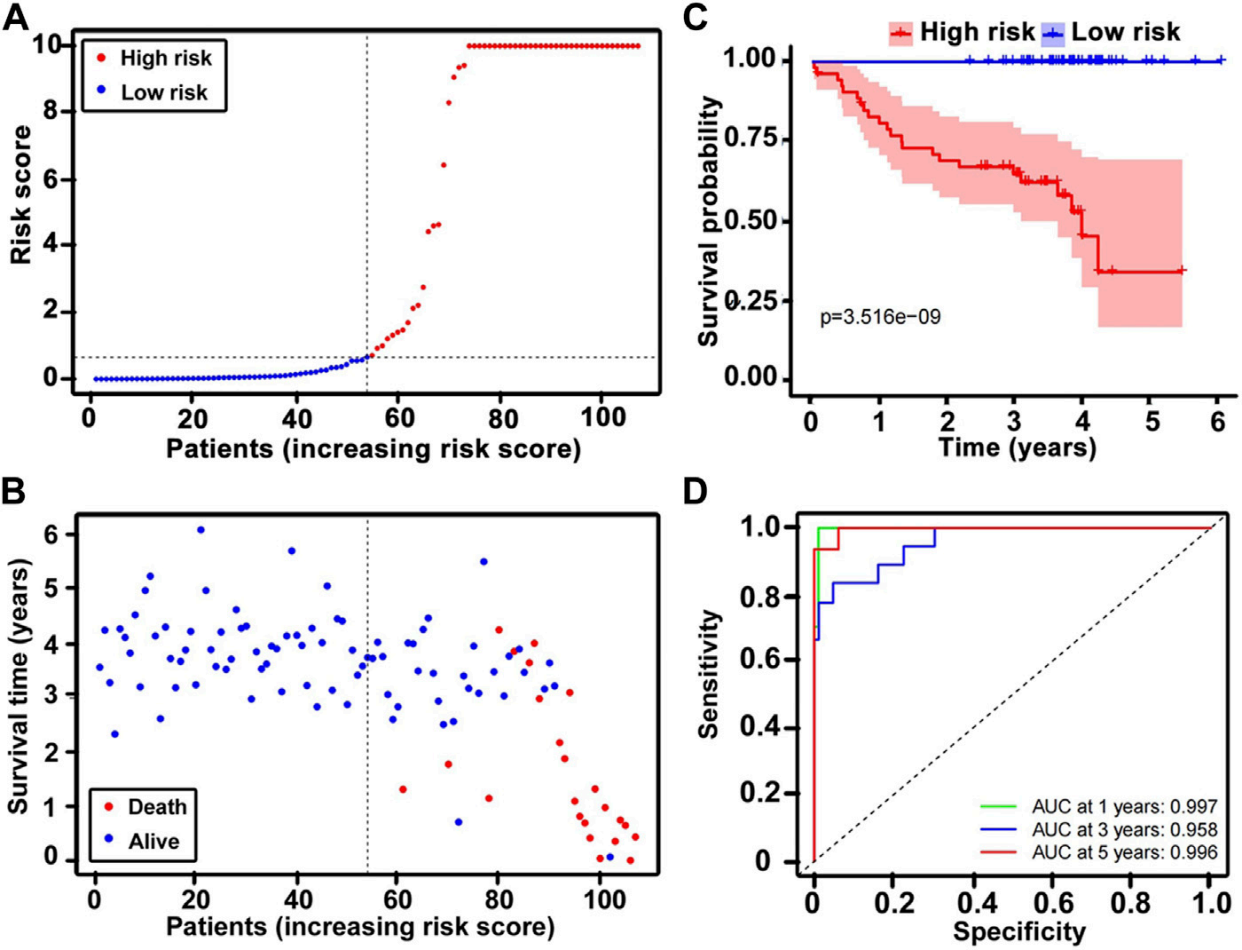
Nine-gene prognostic model for the GSE22762 dataset (N = 107, HG-U133_Plus_2). **(A)** Dot plots comparing the outcomes of subjects in the high- and low-risk cohorts. **(B)** The survival status and time in the high- and low-risk groups. **(C)** K–M survival curves showing the differences between the high- and low-risk groups. **(D)** Time-dependent ROC curve analysis for the prediction survival using the nine-gene model. K–M, Kaplan–Meier; ROC, receiver operating characteristic; AUC, area under the curve.

A total of 107 patients in the training dataset of GSE22762 (HGU-133plus2) were divided into high-risk (risk score > 0.7) and low-risk groups (risk score < 0.7) (Figure 3A). Figure 3B shows that death was more frequently observed in the high-risk group than in the low-risk group. The K–M survival analysis presented a much worse outcome in the high-risk group than that of the low-risk group (log-rank test, *p* = 3.561e-09) (Figure 3C). Also, the AUCs of a time-dependent ROC curve of 1, 3, and 5 years calculated by the nine gene–based risk score model were 0.997, 0.958, and 0.996, respectively (Figure 3D), suggesting that the prediction was highly sensitive and specific. The testing column (GSE22762, N = 44, HGU-133A) verified the predictive values of the nine gene–based risk scores. The K–M curves of the high- and low-risk groups were noticeably different (log-rank test, *p* < 0.05) and the AUCs of 1-, 3-, and 5-year ROC curves were 0.738, 0.679, and 0.628, respectively; these results showed that this prognostic model might be a potential predictor to judge the OS of patients with CLL (Supplementary Figure S3).

GSEA was carried out in two datasets on exploring enriched KEGG pathways in which the analysis suggested that vital enrichment was concentrated in the high-risk cohort, including base and nucleotide excision repair, DNA replication, and valine–leucine and isoleucine degradation (Supplementary Figures S4A,B). Other pathways including homologous recombination, oxidative phosphorylation, mismatch repair, RNA degradation, RNA polymerase, and one carbon pool by folate and lysine degradation were enriched in the high-risk group of the two cohorts.

### The prediction of the nine-gene model on time-to-treatment

**FIGURE 4:**
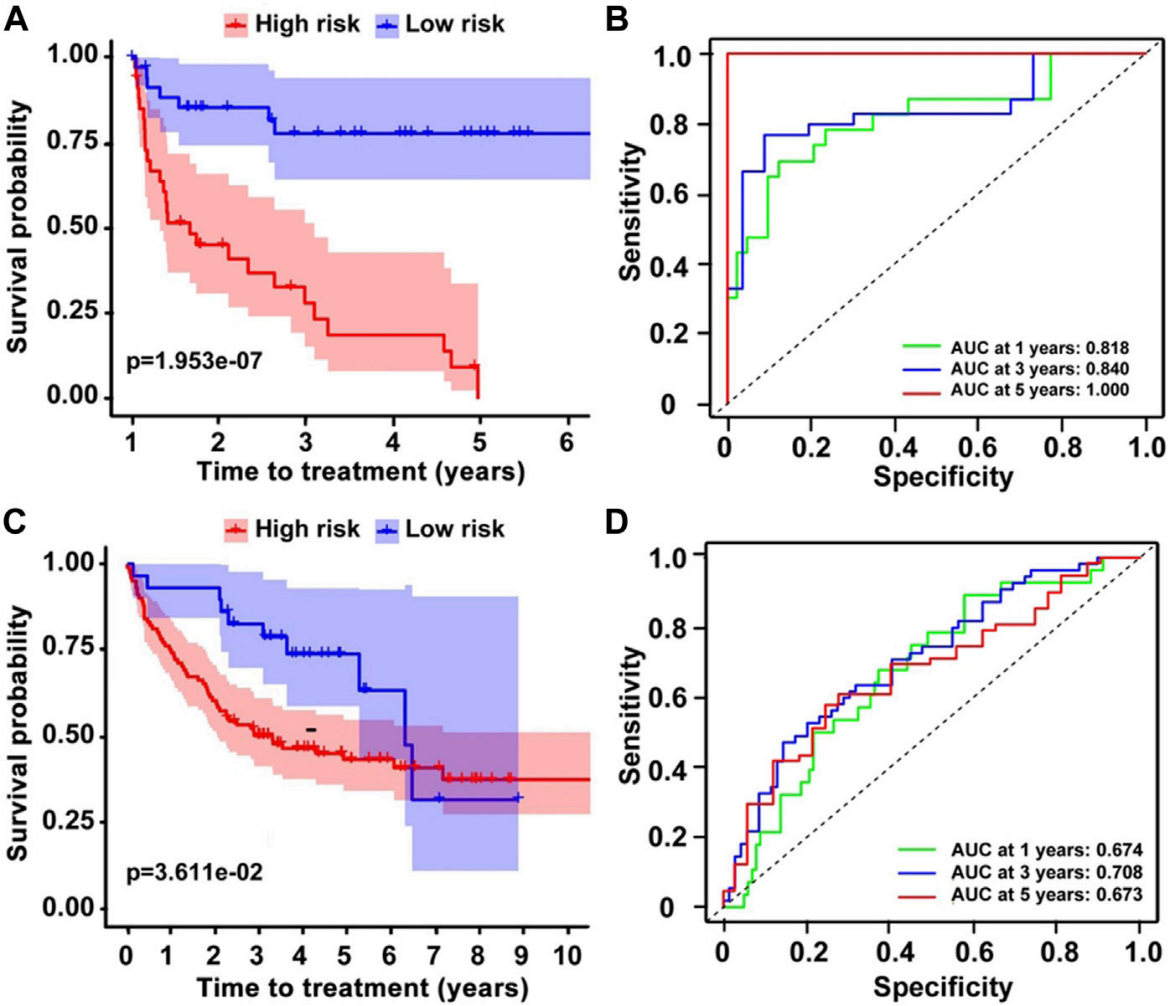
The prediction of TTT on CLL patients. **(A,C)** K–M survival curves showing the different TTT on two datasets and **(B,D)** ROC analysis for the prediction of TTT.

In addition to survival, we also investigated the nine-gene prognostic model on TTT, and the results demonstrated that the nine-gene risk model performed well on predicting TTT in the training dataset (GSE22762). Low-risk patients showed a longer TTT than high-risk patients, and the *p*-value < 0.001 (Figure 4A). Additionally, the time-dependent ROC curve analysis prompted that the AUCs of 1-, 3-, and 5-year TTT were 0.818, 0.840, and 1.000, respectively (Figure 4B). These results were in accordance with testing datasets (GSE39671) (Figures 4C,D), and it indicated that the prognostic model was equally effective in predicting TTT.

### Identification of SE-related hub genes in chronic lymphocytic leukemia using weighted gene co-expression network analysis

**FIGURE 5:**
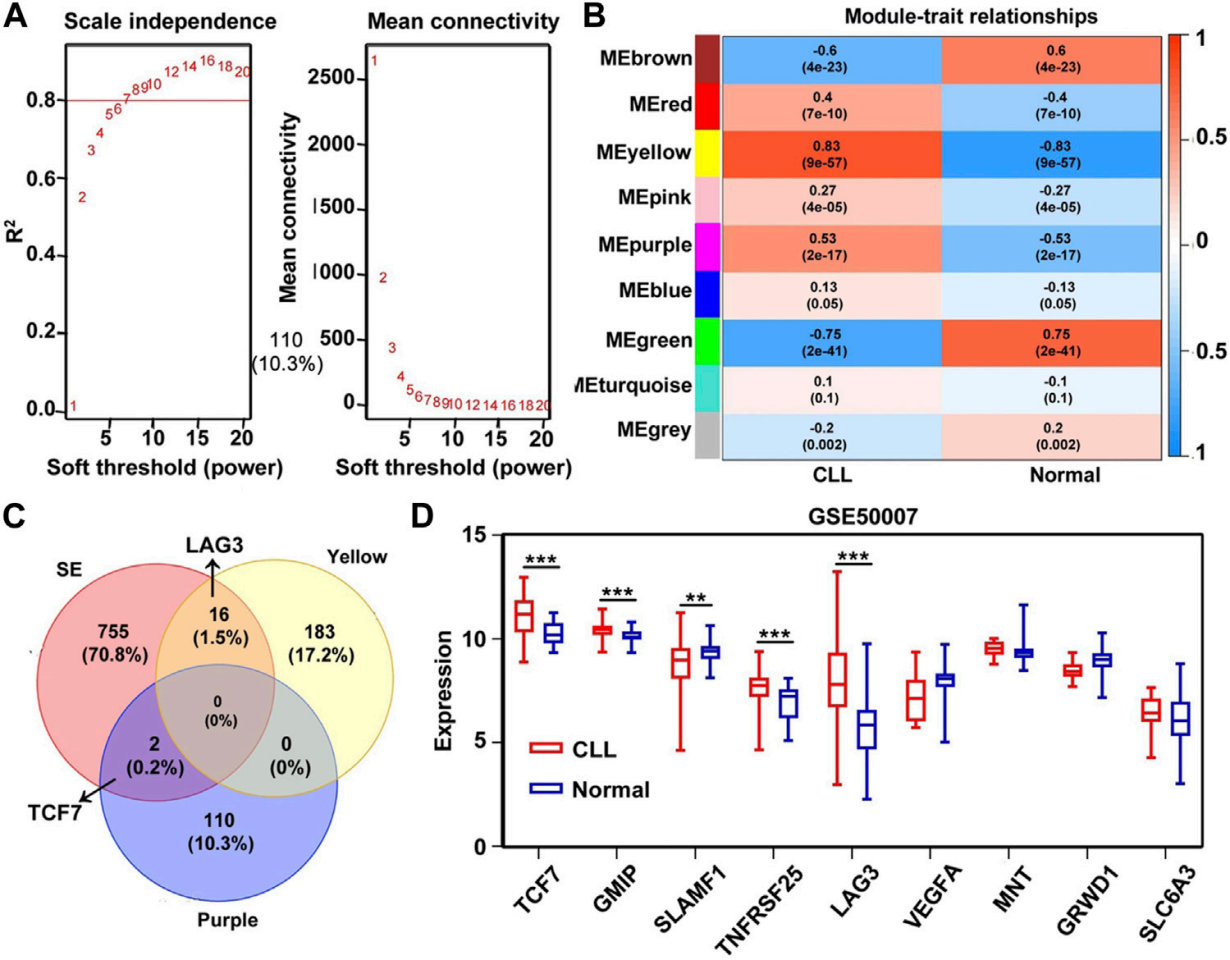
Identification of SE-related hub gene in CLL based on GSE50006 dataset through WGCNA analysis. **(A)** Analysis of the scale independence and mean connectivity (vertical axis) for various soft-thresholding powers (*β* value of horizontal axis). **(B,C)** Heatmap of the correlation between modules and CLL. The yellow and purple module had a high correlation with CLL patients, and the *p*-value in the table specified the correlation. TCF7 and LAG3 appeared in the intersection of SE-related genes and the two modules, respectively. **(D)** The nine hub genes’ expression was significantly different between normal and CLL patients in the GSE50006 dataset. **p < 0.01; ***p < 0.001.

In addition to the prognostic value, we also expected a relationship between the nine-gene model and tumorigenesis. WGCNA was another statistical method for the analysis of finding the different genes between normal and CLL patients. As shown in Figure 5A, the best soft-thresholding value via prediction of the scale independence was *β* = 6. Then, genes were divided into 9 different modules with 9 different colors, and a heatmap was developed according to Pearson’s correlation coefficient (Figure 5B). An intersection between the SE matrix and the nine modules which presented a higher correlation with CLL showed that TCF7 and LAG3 appeared in the interaction genes between module purple, yellow, and SE-associated genes (Figure 5C). Simultaneously, TCF7, GMIP, SLAMF1, TNFRSF25, and LAG3 were found to express differently in normal and CLL patients when we compared the individual expression of nine SE-related hub genes in CLL (Figure 5D). The data indicated that the five genes may play a vital role in regulating the genesis of CLL.

### The validated nine-gene prognostic model and other risk factors

**FIGURE 6:**
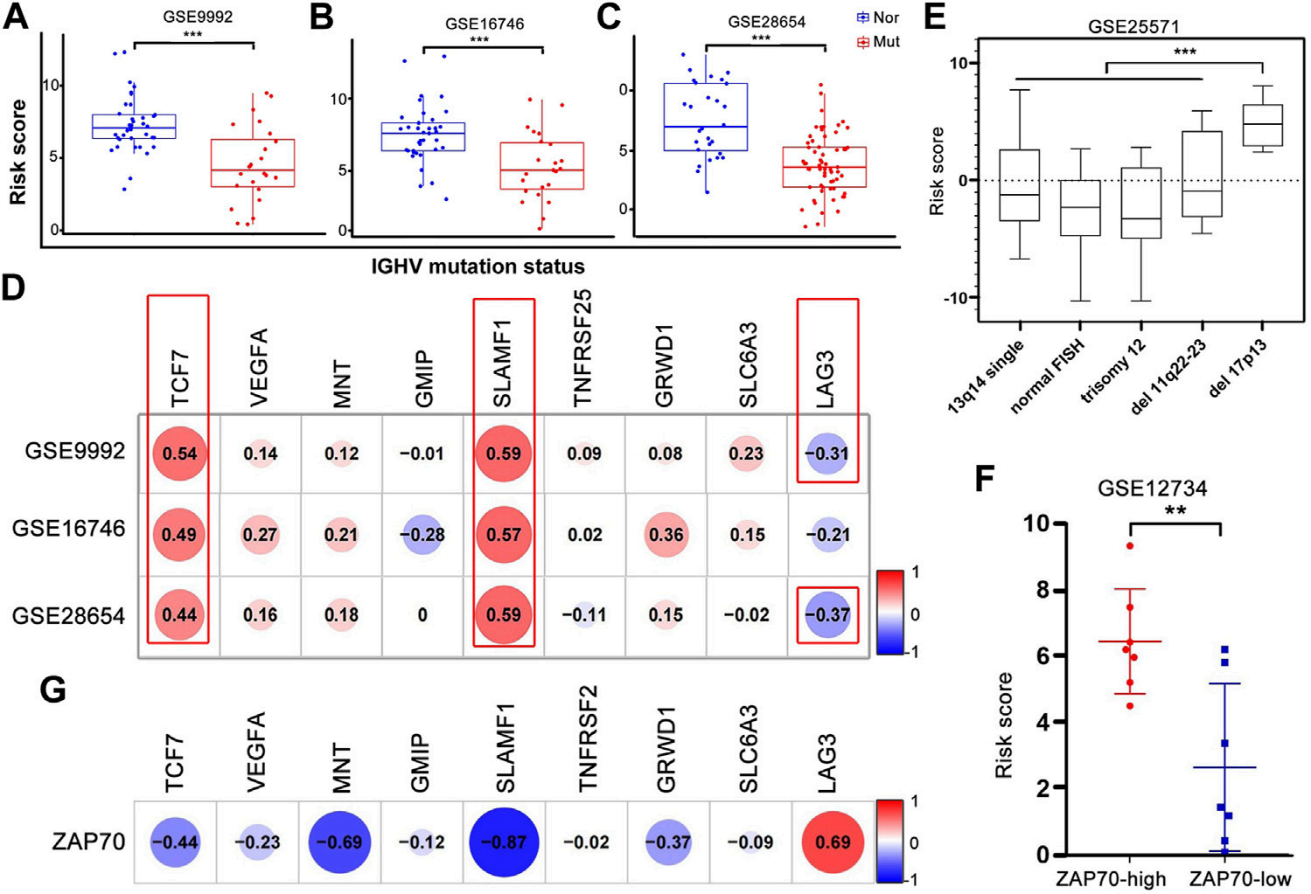
Correlation and variances between the risk score or each gene expression and well-established prognostic markers of CLL. **(A–C)** The risk score of patients with IGHV mutation was significantly lower than that of patients without mutation in GSE9992, GSE16746, and GSE28654. Nor, normal; Mut, mutation. **(D)** The correlation analysis of nine hub genes’ expression and IGHV mutation statue. The *p*-value in red box < 0.001, respectively. **(E)** The risk score of patients with del17p13 was significantly higher than that of other chromosome abnormalities. ***p < 0.001. **(F,G)** The different level of risk score in high- and low-ZAP70 patients and the correlation between nine hub genes’ expression and ZAP70 level.

The performance of the nine-gene prognostic model was additionally evaluated in different subgroups defined by confirmed risk factors. Patients with mutated IGVH genes, 13q14 or single deletion or trisomy 12 on FISH analysis, presented a favorable outcome, whereas patients with unmutated IGVH status, 17p13 or a 11q23 deletions, had an unfavorable prognosis. Unmutated IGHV patients had a higher risk score than mutated IGHV patients in three independent datasets (GSE9992, GSE16746, and GSE28654) (Figures 6A–C). Simultaneously, we analyzed the correlation between IGHV mutation status and each gene in the nine-gene prognostic model. The results reported that the expression of TCF7 and SLAMF1 had a strong positive correlation, and LAG3 showed a negative correlation with IGHV mutation (Figure 6D). Similarly, patients with del17p13 had a higher risk score compared to other chromosome types (*p* < 0.001, Figure 6E). The risk score of ZAP70-high patients was higher than that of ZAP70-low patients; the expression of MNT and SLAMF1 had a negative association, and LAG3 had a positive association with ZAP70, respectively (Figures 6F,G). Additionally, the variation of risk score and each gene expression before and after treatment was provided in Supplementary Figure S5A. The risk score was downregulated after processing with HDAC inhibitory *in vitro*, and VEGFA and MNT were upregulated accompanied by downregulated GMIP and TGFRSF25. In the other two *in vivo* treatment experiments, no significant change was found except LAG3, the LAG3 gene was upregulated consistently after lenalidomide and thalidomide treatment respectively (Supplementary Figures S5B,C).

### Validation of nine-gene prognostic model in ICGC and construction of a nomogram to predict OS

**FIGURE 7:**
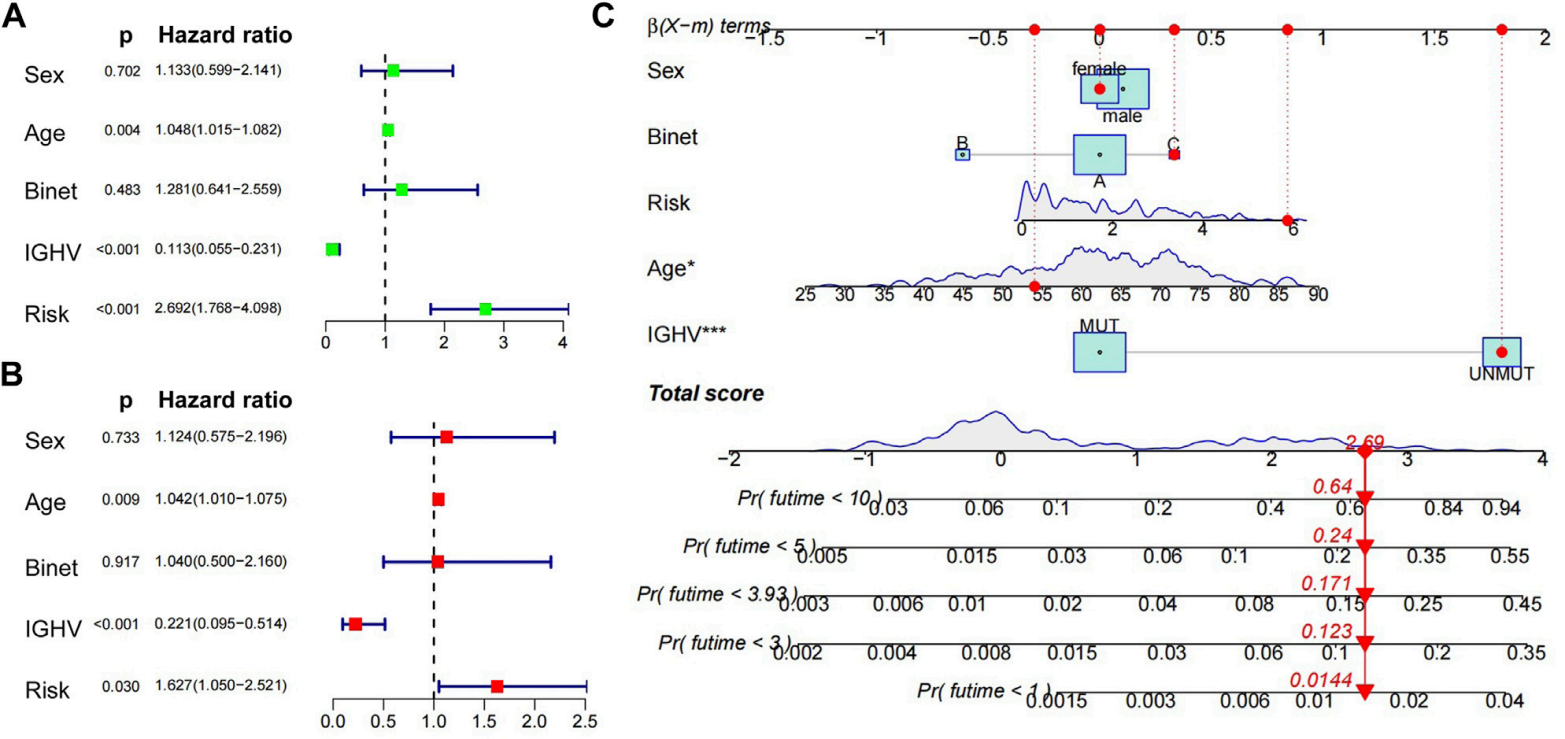
Univariate, multivariate Cox regression analyses and construction of nomograms. **(A) (B)** Univariate and multivariate Cox regression analyses of clinical traits (age, sex, IGHV mutated status, Binet) and nine-gene risk score. **(C)** Nomogram predicting the probability of 1-, 3-, 5-, and 10-year overall survival rates of ICGC-CLL patients. Add the points from these 5 variables together to find the location of the total points. The total points projected on the bottom scales indicate the probability of 1-, 3-, 5-, and 10-year overall survival.

International Cancer Genome Consortium (ICGC, http://daco.icgc.org/), which collected multiple genetic mutations, copy number variants, epigenetic modifications, and clinical data covering 50 tumor types, and we extracted 255 CLL patient data for following analysis. Again, high risk scores were significantly associated with shorter survival time, *p* < 0.001 (Supplementary Figure S6A), and the AUCs of ROC curves of the 3-, 5-, and 10-year survival were 0.731, 0.718, and 0.800, respectively (Supplementary Figure S6B). CLL patients could be divided into two molecular subtypes according to the mutational status of the IGHV, with cases carrying unmutated IGHV (U-CLL) having more aggressive behavior than patients with mutated IGHV (M-CLL). Consistent with the most accepted view, the nine-gene risk score median value was obviously lower in the indolent CLL subtype (M-CLL) compared to the aggressive one (U-CLL) (Supplementary Figure S6D). The nine-gene risk score was associated with the evolution of M-CLL with a median OS of 6.57 versus 8.87 years for patients with high and low risk scores, respectively (*p* = 0.005, Supplementary Figure S6C), while no differences were seen in U-CLL patients in relation to high- and low-risk scores (data not shown). Moreover, on the basis of the obtained sample clinical characteristics, we performed univariate as well as multivariate Cox survival analyses. Age, IGHV mutated status, and risk were identified to be independent prognostic factors for patients with CLL (*p* < 0.05; Figures 7A,B). Based on the nine-gene risk score and clinical traits, a nomogram was constructed to accurately predict CLL patients’ 1-, 3-, 5-, and 10-year survival rates by the using aforementioned clinical indicators and the nine-gene risk score. The C-index of this model was 0.82 (Figure 7C).

### Response of high- and low-risk patients to chemotherapeutic compounds

**FIGURE 8:**
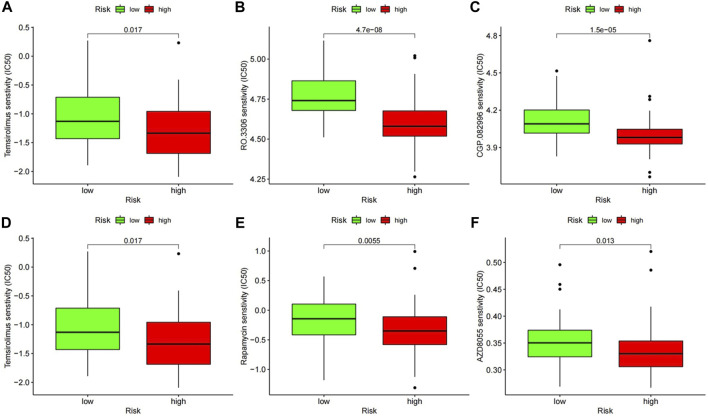
The chemotherapeutic responses of two prognostic subtypes to two kinds of pathway inhibitors. **(A–C)** Inhibitors of mTOR (temsirolimus, rapamycin, and AZD8055). **(D–F)** Inhibitors of CDKs (roscovitine, RO.3306, CGP.082996).

According to the pRRophetic algorithm, we predicted the IC50 of 130 chemotherapeutic agents and pathway inhibitors in both of high- and low-risk patients and found that 25 drugs had lower IC50 in high-risk patients (*p* < 0.05, additional file 1), which indicated that the high-risk patients were more sensitive to these 25 drugs. Among these compounds, some have been reported to have pre-clinical anti-tumor activity in CLL, such as thapsigargin, which was found to be a potent cytotoxin that induced apoptosis by inhibiting the sarcoplasmic/endoplasmic reticulum Ca 2+ ATPase (SERCA) pump, which was necessary for cellular viability. Some have not been reported in CLL before, and therefore the therapeutic effect is still unknown. Interestingly, there were three kinds of compounds which could inhibit the mTOR pathway and CDKs in CLL, respectively, and these have been researched in CLL before and CDK inhibitors have entered clinical trials in patients with relapsed or refractory chronic lymphocytic leukemia. These results could be helpful for the precise treatment of CLL (Figure 8).

## Discussion

CLL is considered to have a highly heterogeneous clinical course, with time to first treatment varying from months to years and many patients eventually progressing and requiring chemotherapy, although initially, CLL is reported as an indolent malignancy. A review of the data so far, disease stratification, IGHV mutation status, 17p, and ZAP70 expression are the validated predictors of overall survival. Beyond that, gene expression analysis was carried out on various surrogate markers for genetic features and prognosis. A total of six surface antigens (CD62L, CD54, CD49c, CD49d, CD38, and CD79b) and prognostic risk models were put in place to diagnose and predict the OS for CLL (Zucchetto et al., 2006). Moreover, some large-scale gene expression profiling analyses generate different prognostic factors (Kienle et al., 2010; Herold et al., 2011a; Schweighofer et al., 2011). But the previous studies constructed no prognostic model according to SE-associated genes which regulate the expression of hub genes related to CLL tumorigenesis.

A super-enhancer is a new concept developed in recent years; a growing body of evidence indicates an explicit relationship between increasing tumorigenesis and malignancy of cancer and SEs. SEs drive not only the expression of genes but also non-coding RNA that regulates biological functions directly and indirectly. LASSO-penalized Cox regression has become popular in recent years because it could minimize overfitting (Ma et al., 2019). Hence, in our article, we use this novel bioinformatic strategy and the Cox proportional hazard regression models to screen and optimize hub genes related to survival.

In our research, the LASSO-penalized Cox regression analysis was carried out by filtering out the potential SE-associated genes and yielding a nine-gene prognostic model to foresee the OS of CLL patients. All of the individual markers in the nine-gene model associated with OS of CLL by Cox regression analysis were identical. K–M survival analysis also indicated that the majority of the nine genes correlated to OS. Beyond that, the nine-gene prognostic model was highly significant in the multivariate analysis of patients without treatment. The AUCs and C-index showed that our model performed well in the prediction of survival. The effectiveness of this prognostic model could be validated by an independent patient cohort. Moreover , this risk model was another indicator of TTT. We utilized the nine-gene risk score in the GSE22762 and GSE39671 datasets, and the results also indicated that the nine-gene model could be applied to predict TTT. The high-risk patients had less time-to-treatment than the low-risk patients. These data strongly indicated that the nine-gene prognostic model was a significant and valid risk forecaster.

We not only evaluated the data by a rigorous training and validation design, but also concentrated on the connection between individual genes and selected disease characteristics, such as IGHV mutation status, FISH abnormality, and ZAP70 expression level. The results of three of the markers (TCF7, SLAMF1, and LAG3) detected according to the association with IGHV status were expected. The lack of a public database that included both survival data and mutation information limited further research on a correlation between the nine-gene model and ZAP70, a FISH abnormality. But in the poor prognosis groups, like ZAP70-high and 17q-patients, the nine-gene risk score was significantly higher than that in the low-risk group, and we found that the low expression of SLAMF1 in CLL was associated with ZAP70-high expression. The quantitative relationship between TCF7, LAG3, and SLAMF1 expression and inferior overall survival was an accurate finding and indicated that these genes had a pathogenic role in CLL. Additionally, the nine-gene prognostic model also played an important role in CLL etiopathogenesis. The WGCNA of the GSE50006 dataset revealed that TCF7 and LAG3 belonged to two gene modules, respectively. In addition to this, the expression of GMIP, SLAMF1, and TNFRSF25 were also significantly different in normal and CLL patients. Therefore, the five genes contained in our model were possibly functionally vital in the pathogenesis of CLL. In the present study, SLAMF1, TCF7, TNFRSF25, MNT, and VEGFA were protective factors, whereas GRWD1, SLC6A3, GMIP, and LAG3 appeared to be harmful factors in CLL; we subsequently discussed each gene in the prognostic model.

Transcription factor 7 (TCF7), the T-cell–specific transcription factor required for T-cell development in animal models, suggests that it probably functions as a tumor suppressor (Roose et al., 1999). TCF7 over-expression in mice led to a disease resembling CLL, indicating that it was probably involved in the CLL transformation in a direct way (Bichi et al., 2002). In CLL, TCF7 expression provided a high rate (74%) of correct assignment of patients at genetic risk (IGHV unmutated, V3-21 usage, 11q-, or 17p-) (Kienle et al., 2010). The aforementioned results are consistent with ours, and this indicates TCF7 plays an important role in CLL.

Signaling lymphocytic activation molecule family member 1 (SLAMF1), also known as CD150, regulates hematopoietic stem cell differentiation, leukocyte adhesion and activation, and humoral immune responses. SLAMF1 comparatively over-express in normal peripheral blood B cells according to the meta-analysis of three gene expression profiling studies. Recently, researchers found lower levels of SLAMF1 expression in cases with ZAP70-high (*p* < 0.001), IGHV-unmutated (*p* < 0.001), and 17q- (*p* = 0.003). In past studies, we believed that loss of SLAMF1 expression in CLL modulates genetic pathways regulating chemotaxis and autophagy and that potentially affects drug responses, suggesting that the effects underlie unfavorable clinical outcomes experienced by SLAMF1-low patients (Bologna et al., 2016). Together, SLAMF receptors, the vital modulators of the BCR signaling axis, improve immune control in CLL by potentially interfering with NK cells (von Wenserski et al., 2021). In our research, the univariate and multivariate analyses presented that downregulated SLAMF1 levels had an independent negative prognostic impact on overall survival (*p* < 0.05). We subsequently discovered that SLAMF1 is relatively overexpressed in IGHV-mutated and ZAP70-low CLL patients. The strict correlation among low levels of it and high-risk genetic features indicated that it probably represented a marker of surrogate genomic complexity; however, the mechanism of this correlation is still unknown.

Lymphocyte activating 3 (LAG3), the immune inhibitory checkpoint receptor, is one of the immunoglobulin superfamily with about 20% amino acid homology with CD4. The expression of it activates and exhausts T, NK cells, B cells, dendritic cells, and regulatory T (Treg) cells. LAG3 high expression in CLL cells correlates with unmutated IGHV (*p* < 0.0001) and decreased treatment-free survival (*p* = 0.0087) (Kotaskova et al., 2010). Increased LAG-3 expression on leukemic cells correlates with shorter time-to-treatment and poor outcome in CLL; moreover, treatment with relatlimab, a novel anti-LAG-3 blocking monoclonal antibody currently under clinical trial for different solid and hematological malignancies including CLL, restored, at least in part, NK and T-cell–mediated anti-tumor responses (Sordo-Bahamonde et al., 2021). CART cell generation with the showing of ibrutinib created enhanced cell viability and expansion of CLL patient-derived CART cells. Also, ibrutinib enriched the mentioned cells with the less-differentiated naïve-like phenotype and declined expression of exhaustion markers (PD-1, TIM-3, and LAG-3) (Fan et al., 2021).

Vascular endothelial growth factor A (VEGFA) is a member of the PDGF/VEGF growth factor family. The angiogenesis process makes a significant contribution to the pathogenesis of B-cell chronic lymphocytic leukemia (B-CLL), the levels of VEGFA and bFGF being higher in patients than in healthy people (Ballester et al., 2020). Whereas, in our research, VEGFA has a protective role in CLL. The high expression of VEGFA indicated a good prognosis by the K–M survival analysis, and in normal samples, the level of VEGFA was higher even though it was not statistically significant.

The TNF receptor superfamily member 25 (TNFRSF25), the receptor expressed preferentially in the tissues of lymphocytes, possibly plays functions vital to the regulation of lymphocyte homeostasis. The receptor stimulates sNF-kappa B activity and regulates cell apoptosis. TNFRSF25 was differentially expressed, activating CLL cells and predominantly detected in those with early clinical stage disease (Cavallini et al., 2015) and probably alters the balance between cell proliferation and death, influencing CLL physiopathology and results in the clinic.

A total of three genes (GRWD1, GMIP, and SLC6A3) have not been described in the context of CLL before, and all of them were upregulated in high-risk CLL patients. The results of the univariate and K–M survival curves were not completely consistent with multivariate analysis. Glutamate-rich WD repeat containing 1 (GRWD1) was identified as one of the ribosomal/nucleolar proteins that promote tumorigenesis (Takafuji et al., 2017). Meanwhile, GRWD1 was also viewed as having histone-binding activity and regulating chromatin openness to specific chromatin locations (Sugimoto et al., 2015). Overexpression in colon carcinoma tissues was related to pathological grading, tumor size, N stage, TNM stage, and poor survival; knockdown of GRWD1 function as an inhibitor on cell proliferation and colony formation, and induced cell cycle arrest and more drug susceptibility, and suppressed the migration and invasion (Zhou et al., 2021). GEM interacting protein (GMIP), a RhoA-specific GAP, in a proteomics screen for proteins interacting with Girdin (Girders of actin), an actin-binding protein critical for neuronal migration to the olfactory bulbs, is identified as one of the major regulators of neuronal migration in the postnatal brain (Ota et al., 2014). Solute carrier family 6 member 3 (SLC6A3) involving in the metabolism of dopamine and catecholamine is the potential gene for Parkinson’s disease and alcoholism. The significance of the aforementioned three genes in CLL remains to be further studied.

In GSE14973, the risk score was significantly downregulated after the valproic acid (VPA) treatment *in vitro*; meantime, protective factors (VEGFA and MNT) were highly expressed, and pathogenic genes (GMIP) were less expressed than in the previous treatment, except TNFRSF25, and these results were almost consistent with our previous conclusion. VPA is a well-tolerated anti-epileptic drug with HDAC inhibitory activity. HDAC1 and HDAC3 inhibition or knockdown results could be figured out in HDAC7 downregulation, which was related to a decline in histone 3 lysine 27 acetylation (H3K27ac) at transcription start sites (TSS) and super-enhancers (SEs) prominently in stem-like BrCa cells. In GSE112953 and GSE15913, the only upregulated gene was LAG3, and it may suggest that combination drug treatment with an anti-LAG3 monoclonal antibody would have a better outcome.

In the present study, a nomogram based on the nine-gene risk score and other clinical traits was constructed, and to determine the predictive effect, we applied the nomogram to a specific patient in the ICGC project; moreover, the predictive model containing the nine-gene risk score was more accurate than the nomogram model containing only four clinical traits. Meanwhile, the risk score was strongly correlated with some known prognostic indicators, such as IGHV mutation state and chromosomal abnormalities. While, a further dissection of the nine-gene risk score on OS in the IGHV mutation state could identify that the nine-gene risk score value was apparent only in the less aggressive M-IGHV subtype, and this predicted trait corresponded to what has been reported in an article which studied the relationship between the ENDOG expression and prognostic study of CLL. The reason why this situation occurred needed further exploration.

The introduction of fludarabine, fludarabine/cyclophosphamide, and either of these combined with rituximab has improved the outcome for younger patients with CLL. Treatment options available for patients in the setting of relapsed disease following receipt of chemoimmunotherapy are limited where most patients have high-risk genomic findings including IgVH un-mutated disease, del (17p13.1) and del (11q22.3) associated with poor treatment response (reviewed in Rassenti et al., 2008). Identifying therapies with novel mechanisms of action for this patient group is important (Johnson et al., 2012). In our research study, all patients were divided into two risk subtypes based on the nine-gene prognostic model, and we endeavored to estimate the drug response of each patient based on IC50 according to the activation of different pathways. ADZ8055 was a dual mTOR kinase inhibitor with inhibition of both mTORC1 and mTORC2 that preferentially decreased cell viability of poor prognostic CLL subsets like with del (11q) or del (17p). One class of drugs that has promise for the treatment of relapsed CLL is the cyclin-dependent kinase (CDK) inhibitors (Seftel et al., 2017). Interestingly, one research study has described that the pan-CDK inhibitor dinaciclib has potent pre-clinical *in vitro* activity against CLL cells independent of high-risk genomic features (Johnson et al., 2012). In our drug sensitivity prediction, there are three kinds of CDK inhibitors which seemed to be more effective for high-risk CLL patients. The reasons that could account for this difference may include: 1) Different drugs have different mechanisms of action, although they are all one class of inhibitor. 2) The criteria of stratifying patients into “High-risk” and “Low-risk” were not consistent. 3) The most important point is the lack of experimental validation in our research.

## Conclusion

To sum up, it was the initial study using the LASSO model to screen prognostic indicators from the profile of SE-associated genes in CLL. A useful prognostic score for OS in untreated CLL patients was presented, and the determination of the score can be achieved via the measurement of the expression levels of nine genes. It also could be done easily in a routine diagnosis. These nine SE-associated genes in this model were not only vital in the development and progression of CLL, but also could assist in guiding the development of alternative treatments.

## Materials and methods

### Data source and microarray analysis

The microarray data and clinical data of GSE22762 (Herold et al., 2011a) and GSE39671 (Chuang et al., 2012), which contain 107 and 130 CLL patients, respectively, were downloaded from the Gene Expression Omnibus (GEO) database. These data were conducted by GPL570 and GPL96/GPL97. Here, 9 other datasets were also analyzed for different purposes, and the details were presented in Table 1 (Fabris et al., 2008; Stamatopoulos et al., 2009a; Stamatopoulos et al., 2009b; Giannopoulos et al., 2009; Mosca et al., 2010; Herold et al., 2011b; Trojani et al., 2011). In the meantime, the International Cancer Genome Consortium (ICGC) CLL sequencing data were extracted from the European Genome-Phenome Database (EGA).

**TABLE 1:** Details of databases used in this research study.

GEO accession	Number	Subgroup of sample	Sample type	Application in article
GSE22762 (Herold et al., 2011a)	151	151 CLL	PBMC	Establishment of a survival model by LASSO and survival analysis of OS and TTT by a nine-gene model
GSE39671 (Chuang et al., 2012)	130	130 CLL	PBMC	Survival analysis of TTT by a nine-gene model
GSE50006	210	188 CLL 32 healthy donors	CD19+ B cells	Validation of expression difference of hub genes between CLL and healthy donors
GSE9992 (Fabris et al., 2008)	60	24 M-CLL 36 U-CLL	CD5+ CD19+ CD23+ B cells	Validation of the correlation of hub gene expression and risk score with IGHV status
GSE16746 (Mosca et al., 2010)	60	23 M-CLL 37 U-CLL	CD5+ CD19+ CD23+ B cells	Validation of the correlation of hub gene expression and risk score with IGHV status
GSE28654 (Trojani et al., 2011)	89	61 M-CLL 28 U-CLL	CD19+ cells	Validation of the correlation of hub gene expression and risk score with IGHV status
GSE25571 (Herold et al., 2011b)	109	FISH abnormality	PBMC	Validation of the correlation of hub gene expression and risk score with genotypic abnormality
GSE12734 (Stamatopoulos et al., 2009a)	14	7 high-ZAP70 7 low-ZAP70	CD19+ cells	Validation of the correlation of hub gene expression and risk score with ZAP70 expression level
GSE14973 (Stamatopoulos et al., 2009b)	28	14 CLL with and without VPA	B cells	Validation of the correlation of hub gene expression and risk score with before and after treatment
GSE112953	22	11 CLL before and after lenalidomide treatment	CD19+ cells	Validation of the correlation of hub gene expression and risk score with before and after treatment
GSE15913 (Giannopoulos et al., 2009)	40	20 CLL before and after thalidomide treatment	PBMC	Validation of the correlation of hub gene expression and risk score with before and after treatment

CLL, chronic lymphocytic leukemia; PBMC, peripheral blood mononuclear cells; M-CLL, IGHV mutated CLL; U-CLL, IGHV un-mutated CLL; OS, overall survival; TTT, time-to-treatment.

### LASSO-penalized cox regression analysis

Super-enhancer–related genes list figured from the primary B-CLL cell was downloaded from SEA version 3.0, which was enriched with a post-translational modification histone mark, H3K27ac ChIP-seq signal. The gene matrix for subsequent analysis was obtained from the overlapping set of genes in the GSE22762 dataset and the SE-associated genes in the primary B-CLL cell. For narrowing and selecting the prognostic genes with potential, the overlapping gene matrix was weighted by the relative coefficients through the LASSO-penalized Cox regression. Ten-fold cross-validation derived the best-fit lambda value to decrease the mean cross-validated error as much as possible via the R package “glmnet”. We chose one median parameter to establish an ideal prognosis model. Then, we measured time-dependent ROC curves and calculated the area under the ROC (AUC).

### Risk score model establishment on predicting patient overall survival

After LASSO-penalized Cox regression analysis was carried out, a risk score model was built using the aforementioned nine genes and could calculate a risk score for each sample through this formula: Risk score = GRWD1 *3.69−TCF7*2.09−VEGFA*0.90−MNT*2.14+ GMIP*1.23−SLAMF1*0.91−TNFRSF25*2.29+ SLC6A3*1.79+LAG3*0.44+8.67 . Patients were separated into high- and low-risk cohorts (median risk score) using the R software “survival” and “survminer” packages, and a *t*-test was used to distinguish death and survival events according to the risk score.

### Cox proportional hazard regression model

Univariate Cox hazard regression analysis validated the correlation among the expression levels of nine genes and OS of each patient by the R package “survival” and “survminer”. At the same time, multivariate Cox hazard regression analyses were performed too. We foresaw the regression coefficient (β-value) and HR. The K–M survival curve and log-rank test of every single gene were also performed by the R package referred previously .

### Weighted gene co-expression network analysis

Weighted gene co-expression network analysis (WGCNA) screened SE-associated hub genes differentially expressed between healthy donors and CLL patients. We counted out the optimal soft-threshold value under the scale independence and mean connectivity analyses. CLL-related genes were clustered into various modules and gained an intersection of significant models and SE-related gene lists via Venn diagrams.

### Gene set enrichment analysis

Under the standard of risk score, we separated the participants into high- and low-risk group sets. Kyoto Encyclopaedia of Genes and Genomes (KEGG) analysis revealed a potential signaling pathway underlying the two sets via gene set enrichment analysis (GSEA v4.1.0 software). *p* < 0.05 and a false discovery rate q < 0.25 were thought to be vital in the statistic.

### Predictive nomogram for prognostic prediction

A nomogram based on independent prognostic factors of clinical traits and the polygenic risk score was constructed to predict the probability of 1-, 3-, 5-, and 10-year OS of patients with CLL. Subsequently, the discrimination of the nomogram was verified using the C-index obtained through a bootstrap method with 1,000 resamples.

### Evaluation of the sensitivity of chemotherapeutic agents

To predict the half-maximal inhibitory concentration (IC50) of chemotherapy drugs in the high- and low-risk groups of CLL patients and to infer the sensitivity of the different patients, we used the “pRRophetic” package in R.

### Statistical analysis

SPSS software vision 25.0 (SPSS, Inc., Chicago, IL, United States) and R software vision 3.6.3 (R Foundation for Statistical Computing, Vienna, Austria) were used to analyze the data in statistics. A two-sided *p* < 0.05 was thought vital in a statistic.

## Data Availability

The datasets presented in this study can be found in online repositories. The names of the repository/repositories and accession number(s) can be found in the article/Supplementary Material.
