# Supplementary figures and images for: Super-Enhancer–Associated nine-gene prognostic score model for prediction of survival in chronic lymphocytic leukemia patients
Source: Front Genet. 2022 Sep 15;13:1001364. doi: 10.3389/fgene.2022.1001364 (PMC9521409; doi:10.3389/fgene.2022.1001364)

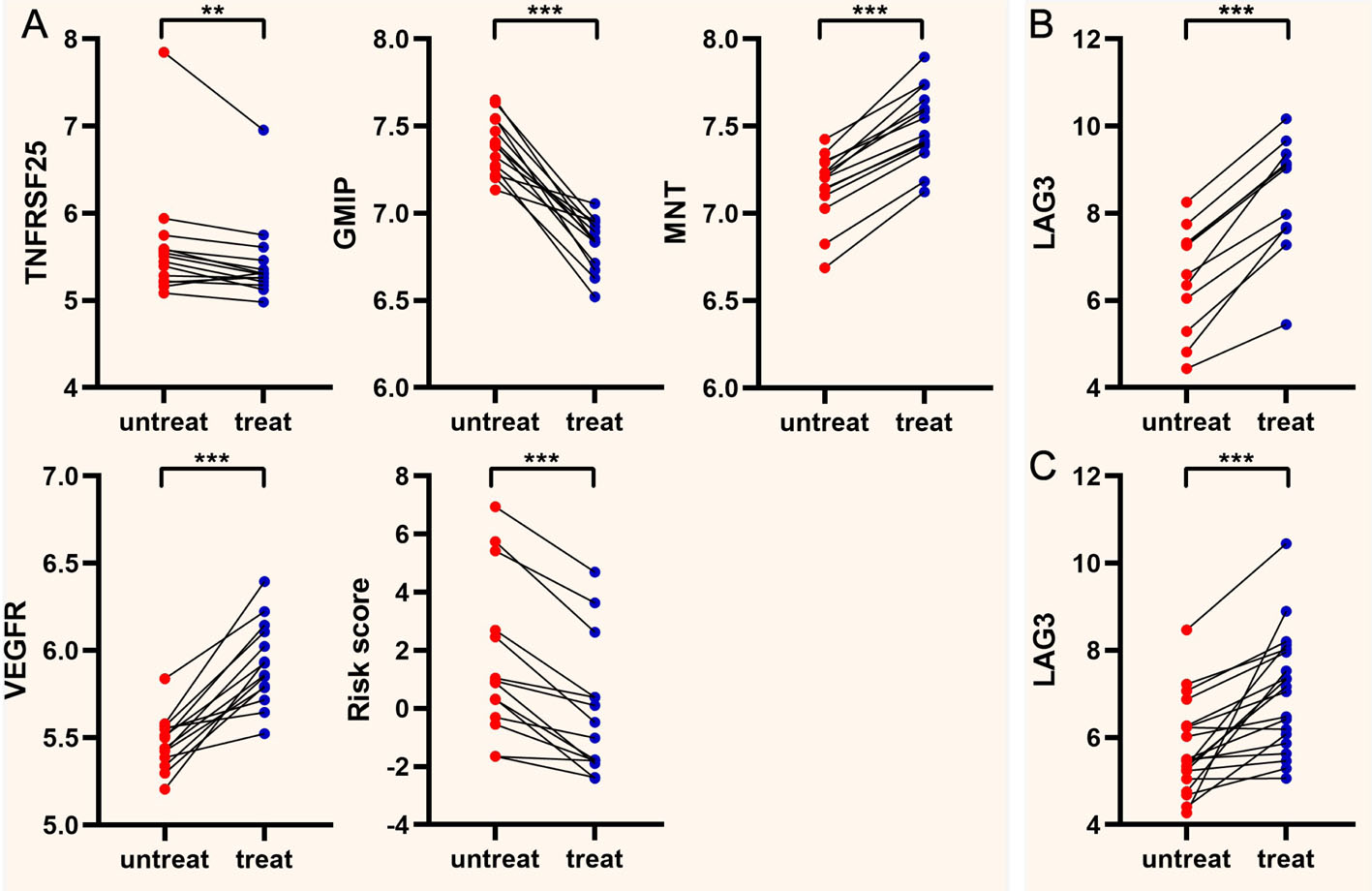

Supplement: Supplementary file 1 [file Image5.jpg]

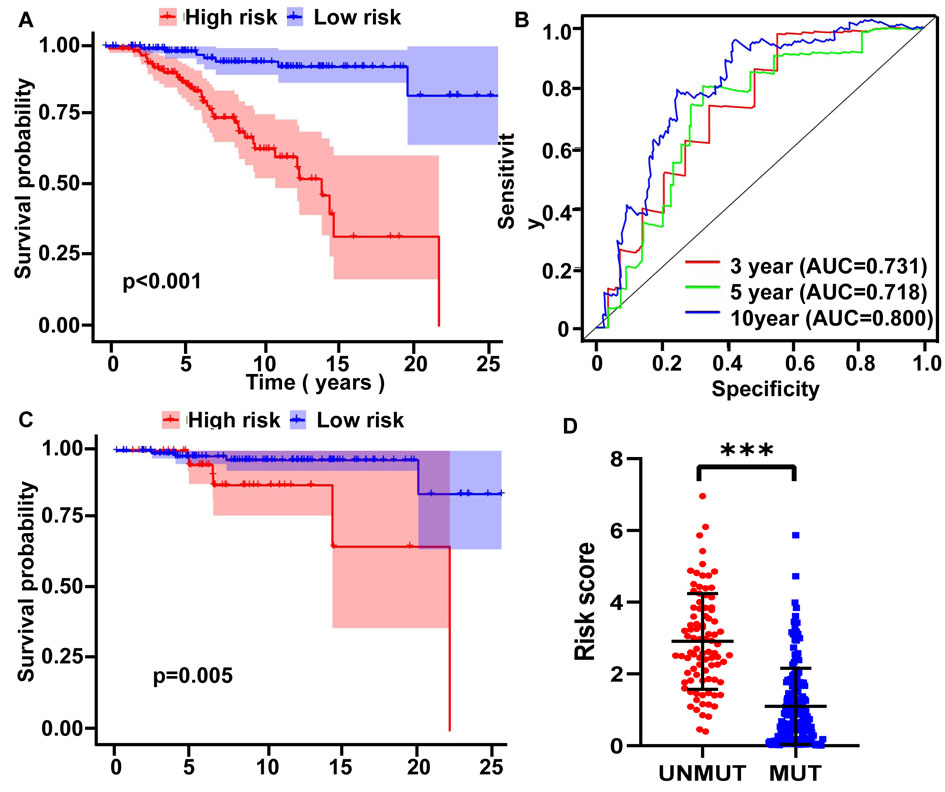

Supplement: Supplementary file 2 [file Image6.jpg]

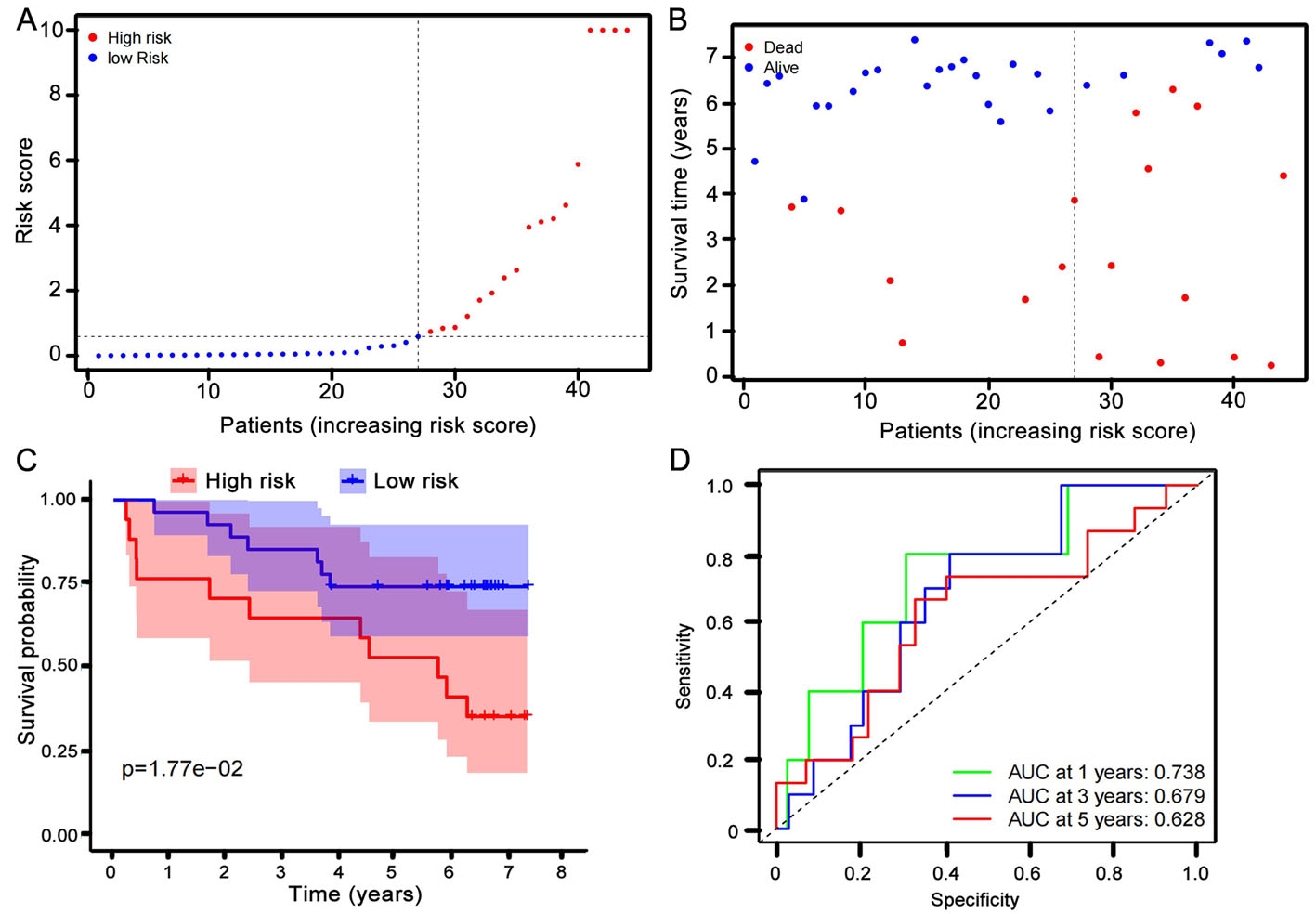

Supplement: Supplementary file 3 [file Image3.jpg]

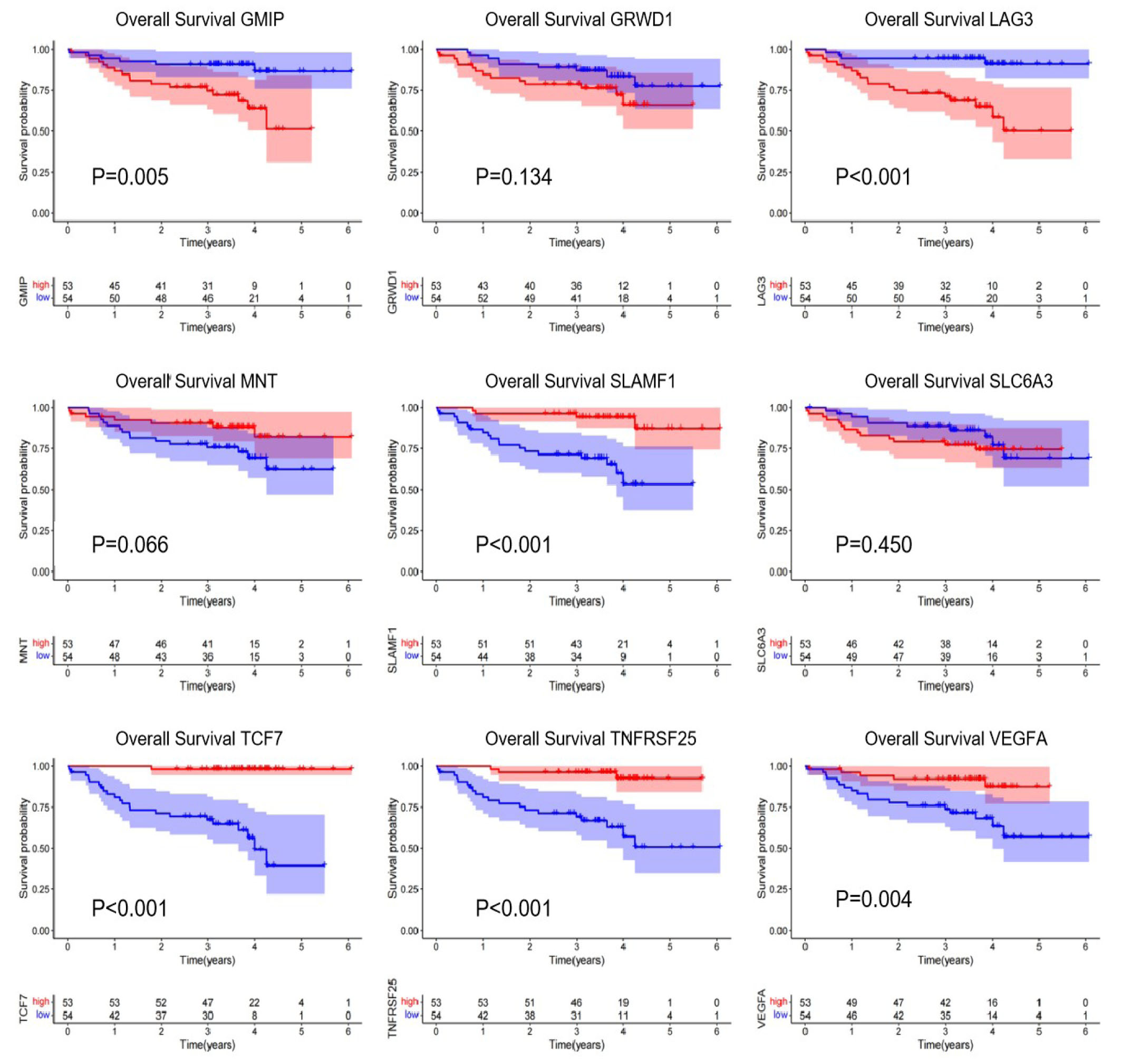

Supplement: Supplementary file 4 [file Image2.jpg]

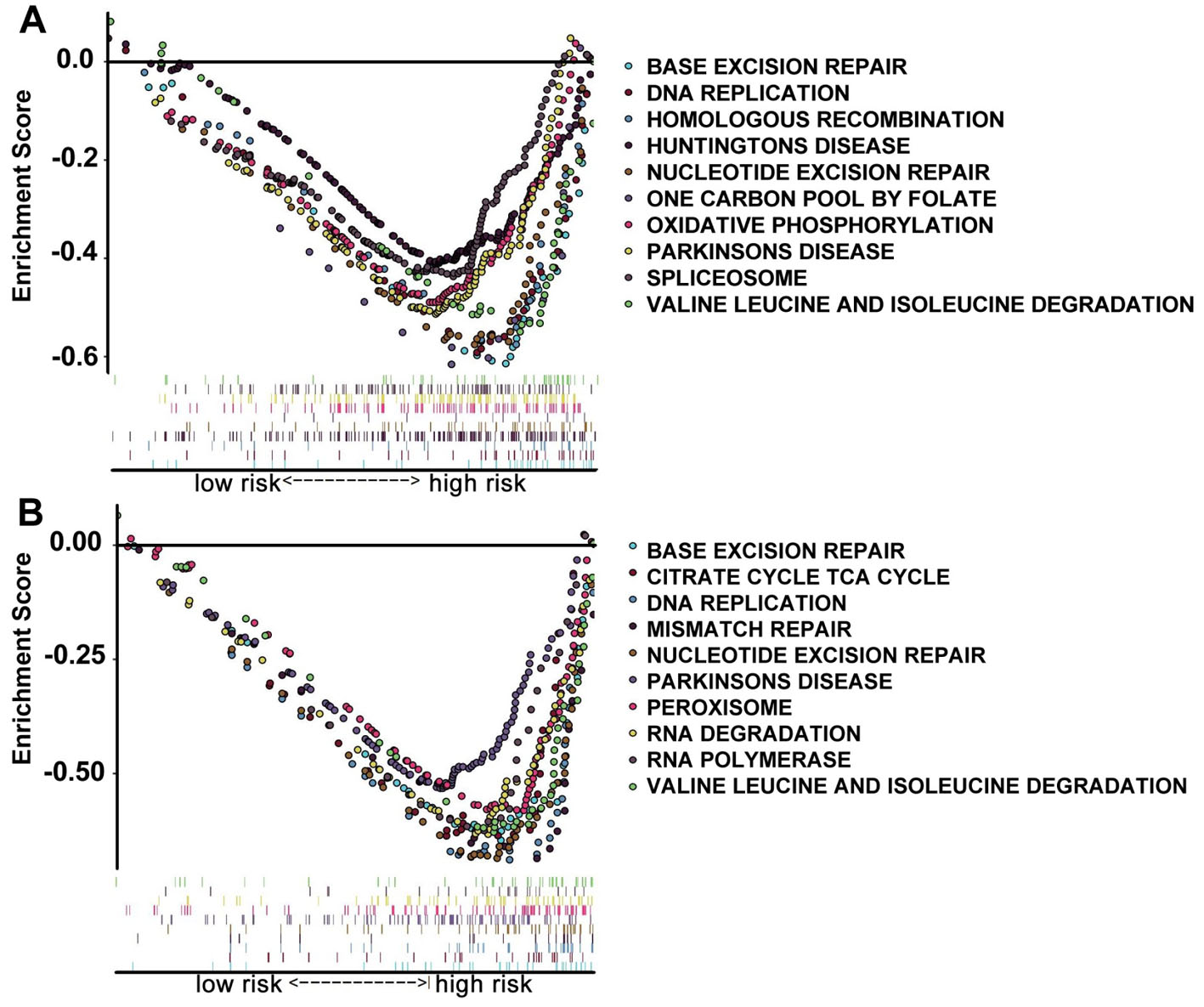

Supplement: Supplementary file 5 [file Image4.jpg]

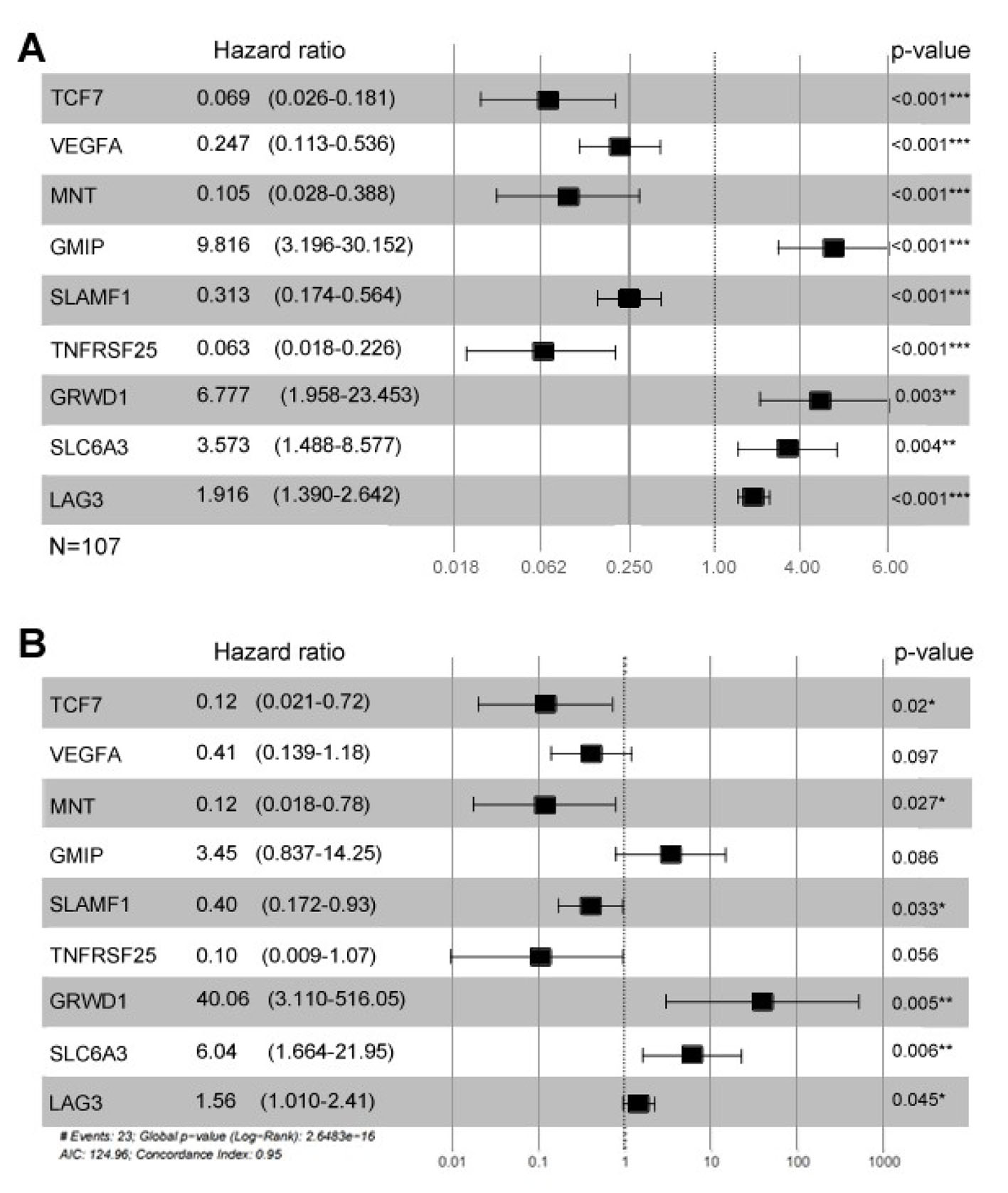

Supplement: Supplementary file 6 [file Image1.jpg]
